# Cigarette Smoking Promotes Inflammation in Patients with COPD by Affecting the Polarization and Survival of Th/Tregs through Up-Regulation of Muscarinic Receptor 3 and 5 Expression

**DOI:** 10.1371/journal.pone.0112350

**Published:** 2014-11-06

**Authors:** Ming-Qiang Zhang, Yong Wan, Yang Jin, Jian-Bao Xin, Jian-Chu Zhang, Xian-Zhi Xiong, Long Chen, Gang Chen

**Affiliations:** 1 Department of Respiratory and Critical Care Medicine, Key Laboratory of Pulmonary Diseases of Health Ministry, Union Hospital, Tongji Medical College, Huazhong University of Science and Technology, Wuhan, China; 2 Department of Respiratory and Critical Care Medicine WUHAN NO. 1 HOSPITAL, Wuhan, China; Seoul National University College of Pharmacy, Republic of Korea

## Abstract

**Background:**

CD4^+^ T cells in the lung are involved in the pathogenesis of chronic obstructive pulmonary disease (COPD), although CD4^+^ T cell subsets and the direct effect of smoking on these cells, especially the expression of MRs, have not been comprehensively examined.

**Methods:**

First, circulating CD4^+^ T cell subsets in healthy nonsmokers, patients with SCOPD and patients with AECOPD were evaluated by flow cytometry. Then, differentiation experiments were carried out using RT-PCR, and Ki-67/Annexin V antibodies were used to measure proliferation and apoptosis. We also explored the impact of CSE on the differentiation and survival of CD4^+^Th/Tregs and examined the expression of MRs in healthy nonsmokers and patients with SCOPD.

**Results:**

We found the percentages of circulating Th1 and Th17 cells were increased in patients with AECOPD, while the percentage of Th2 cells was decreased in patients with SCOPD. The percentages of Th10 cells were decreased in both patients with SCOPD and patients with AECOPD, while the percentages of Tregs were increased. In addition, the percentages of CD4^+^α-7^+^ T cells were decreased in patients with SCOPD and patients with AECOPD. However, only the decrease observed in patients with AECOPD was significant. In vitro studies also revealed MR expression affected the polarization of T cells, with different CD4^+^ T cell subtypes acquiring different MR expression profiles. The addition of CSE facilitated CD4^+^ T cell polarization towards pro-inflammatory subsets (Th1 and Th17) and affected the survival of CD4^+^ T cells and Treg cells by up-regulating the expression of MR3 and 5, resulting in an imbalance of CD4^+^ T cell subsets.

**Conclusions:**

Our findings suggest an imbalance of circulating CD4^+^ T cell subsets is involved in COPD pathogenesis in smokers. Cigarette smoking may contribute to this imbalance by affecting the polarization and survival of Th/Tregs through the up-regulation of MR3 and MR5.

## Introduction

Chronic obstructive pulmonary disease (COPD) is characterized by persistent airflow limitation and progressive airway inflammation, and its prevalence is rapidly increasing worldwide. Inflammation in the airways is triggered by inhalation of hazardous gases and particles; tobacco smoking is the leading contributing factor for this type of inflammation [1]. Chronic smoking can lead to refractory inflammation in the lung, which eventually results in destruction of the alveolar space, loss of surface area for gas exchange and loss of elasticity (i.e., emphysema) [2]. However, the mechanisms underlying these changes following lung exposure to cigarette smoke have not been completely elucidated.

Increasing evidence indicates that adaptive immune responses are involved in the pathogenesis of COPD, and inflammation mediated by T cells has specifically been identified as a key component [3]. Although several studies have focused on CD4^+^ T cells in the blood of patients with COPD [4], [5], there are few comprehensive examinations of circulating CD4^+^ T cell subsets in this disease. Recent research has shown that soluble components extracted from cigarette smoke (CSE) could significantly reduce T cell activation, proliferation and the expression of cytotoxic proteins, such as granzyme-B [6], thereby suppressing dendritic cell functions and favoring the development of T helper (Th)2 immunity [7]. However, other types of T cells, particularly the Th1 and Tc1 subsets, are present in the airways and parenchyma of smokers with COPD [8]. Thus, the precise influence of CSE on CD4^+^ T cells, particularly whether cigarette smoke suppresses or facilitates the function and proliferation of these cells, remains unclear.

Recent emerging studies on the non-neuronal cholinergic system have shown that the cholinergic system is implicated in many diseases, such as arthritis, angiogenesis, cancer, non-healing wounds and inflammation [9]. Lymphocytes have been shown to both express cholinergic receptors, including muscarinic acetylcholine receptors (mAChRs), and serve as a source of Ach [10]. Indeed, accumulating evidence has further indicated that T cell-synthesized ACh acts as an autocrine and/or paracrine factor via ACh receptors on immune cells to modulate immune function [11]. COPD is a chronic inflammatory disease that is characterized by hyperfunction of the cholinergic system [12]. However, whether the cholinergic system is involved in the pathogenesis of COPD through the regulation of T cells remains unknown. In particular, whether smoking affects CD4^+^ T cells through the cholinergic system, whether CSE enhances the expression of mAchR in CD4^+^ T cells, and whether the effect of smoking could be decreased by blocking the mAchR are questions that have remained unanswered in the field.

To answer these questions, we examined and compared circulating CD4^+^ T cell subsets (Th1, Th2, Th17, Tregs, Th10, and CD4^+^α-7^+^ T cells) in healthy nonsmokers, patients with stable COPD, and patients with acute exacerbation in COPD. Then, in vitro experiments were carried out to investigate the effects of smoking and the muscarinic receptor (MR) signaling system on the differentiation and survival of CD4^+^ Th/Tregs. Our results identified an imbalance of pro/anti-inflammatory CD4^+^ T cell subsets in patients with COPD. Moreover, CSE affected the differentiation and survival of Th/Tregs through the up-regulation of MRs, resulting in an imbalance of Th/Tregs and the development of chronic inflammation in patients with COPD.

## Materials and Methods

### Subjects

The study was approved by Ethics Committee of the Tongji Medical School, Huazhong University of Science and Technology. All patients and volunteers were informed of the research process and signed informed consent forms. Based on the Global Initiative for Chronic Obstructive Lung Disease (GOLD) criteria [13], 24 patients with stable COPD (SCOPD), 14 patients with acute exacerbation COPD (AECOPD) (clinical characteristics listed in Table 1), and 14 healthy nonsmokers were enrolled (no smoking; age, sex, matched). Patients with AECOPD were diagnosed as the initiation of exacerbated COPD symptoms, which required hospitalization, in the previous 72 h without any new therapeutic intervention. The following examinations or tests were performed: medical history, physical examination, routine blood tests, liver and kidney functions tests, serum electrolytes and pulmonary function. Additionally, a chest X ray or helical computed tomography (CT) and electrocardiography (ECG) or heart ultrasound examination were performed to exclude other diseases. Peripheral blood samples were collected from all patients and volunteers.

**Table 1:** Demographic and spirometric values of healthy nonsmokers, patients with SCOPD and patients with AECOPD.

	Healthy nonsmokers	SCOPD	AECOPD
**Participants**	14	24	14
**Age (years)**	65.6±7.8	66.5±7.2	69.1±9.6
**Sex ratio (M/F)**	12/2	19/5	11/3
**Smoking history (pack, y)**	0	41.9±17.6*	47.2±19.5*
**FEV1 (% predicted)**	104.6±10	47.7±24.2*	35.1±15.1*^#^
**FEV1/FVC (%)**	85.2±6.1	48.8±15.2*	47.9±10.1*

All data are presented as the mean±SD. FEV1: Forced expiratory volume in one second, measured post bronchodilatation and FVC: Forced vital capacity. *P<0.05 compared with healthy nonsmokers; ^#^P<0.05 compared with SCOPD patients.

### Sample collection and isolation of peripheral blood T (PBT) cells

Peripheral blood samples were collected in heparin-treated tubes from each subject within 24 h of arrival to the hospital or during the medical examination for healthy nonsmokers. The blood samples were immediately immersed in ice and then centrifuged at 1,200×g for 5 min. After the peripheral blood mononuclear cells (PBMCs) were isolated from heparinized blood by Ficoll-Hypaque gradient centrifugation (Pharmacia, Uppsala, Sweden), PBT cells were recovered from the non-adherent cells after 24 h of PBMC culture, as previously described [14], [15], [16]. The recovered PBT cell fraction was greater than 85% CD3^+^ cells, as assessed by flow cytometry. Isolated PBT cells were resuspended in complete medium RPMI 1640 (HyClone) plus 10% FBS (HyClone) and then placed in an incubator at 37°C in 5% CO_2_ for subsequent experiments.

### Flow cytometry

Surface marker expression and intracellular cytokine production by T cells were determined using flow cytometry. The staining was performed using anti-human-specific antibodies (Abs) conjugated with the following fluoresceins: isothiocyanate (FITC), phycoerythrin (PE), peridinin chlorophyll protein (PerCP)-cy5.5, allophycocyanin (APC), PE-CY7, or Alexa Fluor 647. These human Abs included anti-CD3, anti-CD4, anti-CD8, anti-CD25, anti-Foxp3, anti-IL-17A, anti-IL-10, anti–IFN-γ, anti-IL-4, and anti-Ki-67 mAbs, as well as isotype mAbs, which were purchased from BD Biosciences or eBioscience (San Diego, CA). The expression of nicotine receptor α7 on T cells was detected by its binding the alpha-bungarotoxin (Invitrogen, USA). Intracellular staining for IL-17-, IL-10-, IFN-γ-, and IL-4–producing T cells was performed on T cells stimulated with PMA (50 ng/ml; Sigma-Aldrich, St. Louis, MO) and ionomycin (1 µM; Sigma-Aldrich) in the presence of GolgiStop (BD Biosciences) for 5 h. Intracellular cytokines were then stained with the corresponding mAbs conjugated to fluoresceins after fixation and permeabilization (permeabilization kit, eBioscience) according to the manufacturer's instructions. Isotype controls were included to enable the correct compensation and confirm antibody specificity. Flow cytometry was performed using a fluorescence-activated cell sorter (FACS) Canto II (BD Biosciences) with BD FCSDiva software and FCS Express 4 (De Novo Software) software.

### Preparation of CSE

Our preparation of CSE was performed according to the method of Blue and Janoff [17]. CSE was prepared by drawing cigarette smoke into a 50-ml plastic syringe and then slowly bubbling the smoke into a tube that contained 5 ml of sterile RPMI 1640 medium. Each 5 ml of CSE was produced using two cigarettes (Huang Helou, Wuhan China), and the concentration of the original CSE was considered to be 100%. All materials were sterile and used only once. Then, the solution of CSE was filtered through 0.22- µm filters. To ascertain the vitality of the substances in CSE, all CSE preparations were made within half an hour prior to each experiment. The concentrations of CSE used in the experiments were chosen according to the concentration gradient experiment.

### Real-time quantitative PCR

Total RNA was prepared from T cells using TRIzol reagent (TaKaRa). In all, 2 µg of total RNA was reverse transcribed into 20 µl of complementary DNA using the cDNA reverse transcription kit (TaKaRa). Melting curves were generated to establish the purity of the amplified band after 40 cycles of 30 s at 94°C, 30 s at 57°C, and 30 s at 72°C. Amplified fragments of the expected size were analyzed with a 2% agarose gel and were photographed under UV light. Q-PCR was performed on a StepOnePlus Real-Time PCR System (Applied Biosystems) in a 10- µl reaction that contained 1 µl of cDNA and SYBR Premix Ex Taq (TaKaRa). The following PCR parameters were used: 95°C for 3 min; 40 cycles of 95°C for 10 s and 60°C for 30 s; and a melting curve from 65°C to 95°C in increments of 0.5°C for 5 s. The expression level of GAPDH was used as an internal control. The relative expression was calculated with the comparative Ct method using StepOne Software v2.1 (Applied Biosystems) and was expressed as the fold change compared to the control. The specific primer pairs used to amplify genes are listed in Table 2.

**Table 2:** Real-time RT-PCR primer sequences.

Target gene	Sequence (5′—3′)
**TBX21 (T-bet)**	F:TGGTCCAAGTTTAATCAGCACCAG
	R:CCCGGCCACAGTAAATGACAG
**GATA3**	F:GAGATGGCACGGGACACTAC
	R:GGTCTGACAGTTCGCACAGG
**RORC**	F:CTGCAAGACTCATCGCCAAAG
	R:TTTCCACATGCTGGCTACACA
**FOXP3**	F:CTGGCAAATGGTGTCTGCAAGT
	R:CTGCCCTTCTCATCCAGAAGATG
**GAPDH**	F:GCACCGTCAAGGCTGAGAAC
	R:TGGTGAAGACGCCAGTGGA
**MR1**	F:AGGAAGTCAGGAGCCAGCAG
	R:GCACCATCTCACACCGCAATC
**MR2**	F:GTCAGAATGGAGATGAAAAGCAGA
	R:GAAAGCCAACAGAATAGCCAAGA
**MR3**	F:TCCGAGCAGATGGACCAAGA
	R:GAAGCTTGAGCACGATGGAGTAGA
**MR4**	F:AGATGGCAGGCCTCATGATTG
	R:CTGGGTTGGACAGGAACTGGA
**MR5**	F:ACAAGAGGAAGCACACTGGGTAA
	R:GCTGGTTCTCACTGGCACAAG

All of these primers were synthesized by TaKaRa in Dalian.

### Differentiation and polarization of T cell subsets

PBT cells (1×10^6^) were cultured in 300 µl of complete medium in 96-well plates, and soluble anti-CD3 (1 µg/ml) and anti-CD28 Abs (1 µg/ml)(both from eBioscience) were added to achieve TCR stimulation via the TCR/CD3 complex. The cells were cultured in RPMI 1640 supplemented with 10% fetal calf serum in a 5% CO_2_ humid atmosphere at 37°C for 5 days. To investigate the influence of cigarette smoke and the cholinergic system on the differentiation of T cells, CSE (0.33% = 1 µl of 100% CSE in 300 µl of medium) and an MR agonist or antagonist were added to the medium. The mAChRs were activated with muscarine (50 µM, the dose discussed in other papers was not suitable for our experimental conditions; this dose was selected based on a preliminary experiment, and toxicity and side effects were not observed at this dose). The mAChRs were inhibited with atropine (100 µM) from Sigma-Aldrich or Tocris. To derive Th1/Th2/Th17/Treg cells, the following exogenous cytokines were added: 20 ng/ml IL-12 for Th1; 4 ng/ml IL-4 for Th2; 20 ng/ml IL-6 and 5 ng/ml TGF-β1 for Th17; and 2 ng/ml IL-2 and 5 ng/ml TGF-β1 for Treg. All of the aforementioned recombinant human cytokines were purchased from Peprotech.

### Proliferation and apoptosis of T cells

PBT cells (1×10^6^) were cultured in 300 µl of complete medium in 96-well plates. The cells were cultured in RPMI 1640 supplemented with 10% fetal calf serum in a 5% CO_2_ humid atmosphere at 37°C. PBT cells were cultured in the presence of medium alone, CSE or mAChR agonist/antagonist. The mAChRs were activated with muscarine (50 µM) and inhibited with atropine (100 µM) (Sigma-Aldrich or Tocris). In the proliferation assays, 1 µg/ml PHA and 50 ng/ml PMA (both from Sigma-Aldrich, St. Louis, MO) were added to the medium to stimulate T cell proliferation. After 5 days, the cells were harvested and then stained with APC-conjugated Annexin V and propidium iodide (Annexin V Apoptosis Detection Kit APC; eBioscience) at room temperature in the dark for 10 min. Finally, the proportion of apoptotic T cells was determined by flow cytometry. For T cell proliferation assays, intracellular staining with Alexa Fluor 647 conjugated anti-human Ki-67 (eBioscience) was performed.

### Statistics

The data are expressed as the mean ± SD (unless indicated in the figure legends). Comparisons of the data between different groups were performed using a Kruskal-Wallis one-way analysis of variance (ANOVA) with Tukey and Dunn post-hoc tests for between-group comparisons. Comparisons between healthy nonsmokers and patients with SCOPD in the proliferation and apoptosis assays were performed using the Mann-Whitney U test. Data analysis was performed with GraphPad Prism V5.01 software (GraphPad Software, La Jolla, California), and two-tailed P-values of less than 0.05 were considered statistically significant.

## Results

### 1 Changes in CD4^+^ T cell subsets in the three patient populations

#### 1.1 The percentages of Th1 and Th17 cells are increased in patients with AECOPD, while the percentage of Th2 cells is decreased in patients with SCOPD

**Figure 1:**
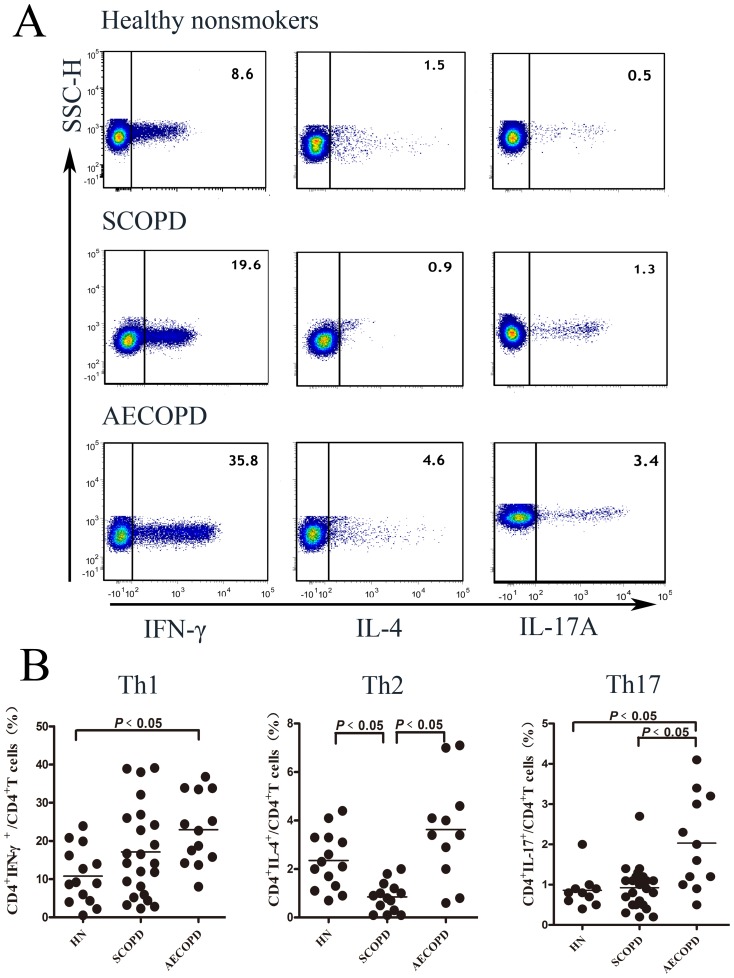
The percentages of Th1 and Th17 cells are increased in patients with AECOPD, while the percentage of Th2 cells is decreased in patients with SCOPD. (**A**) Representative flow cytometric dot-plots of Th1, Th2 and Th17 cells (*Th1: CD3^+^CD8^−^IFN-γ^+^ T cells, Th2: CD3^+^CD8^−^IL-4^+^T cells, Th17: CD3^+^CD8^−^IL-17A^+^T cells*) in healthy nonsmokers (HN), patients with SCOPD and patients with AECOPD are shown. (**B**) Comparisons of the percentages of Th1, Th2 and Th17 cells in healthy nonsmokers, patients with SCOPD and patients with AECOPD (*n = 14, 24, 14, respectively*) are shown. Horizontal bars indicate the mean value. The comparisons were made using a Kruskal-Wallis one-way ANOVA on ranks.

To investigate the changes in pro-inflammatory CD4^+^ T cells in COPD, we measured and analyzed the levels of Th1 cells (CD3^+^CD8^−^IFN-γ^+^), Th2 cells (CD3^+^CD8^−^IL-4^+^), and Th17 cells (CD3^+^CD8^−^IL-17A^+^) in the blood among the three patient groups (Figure 1A). Due to the limited quantity in some samples, not all CD8^+^ T cell subsets were detected.

As shown in Figure 1B, the percentages of Th1 and Th17 cells were remarkably elevated in patients with AECOPD (22.73±9.14%; 2.03±1.16%, respectively) compared to healthy nonsmokers (10.80±7.34%; 0.75±0.42%, respectively). There were no significant changes in the percentages of these cells in patients with SCOPD (17.15±12.28%; 0.86±0.44%, respectively). In addition, there were more Th17 cells in patients with AECOPD compared to patients with SCOPD (P<0.05). In addition, the percentage of Th2 cells was significantly reduced in patients with SCOPD (0.85±0.61%) compared to healthy nonsmokers (2.30±1.10%); however, no difference was observed was in patients with AECOPD (3.63±2.13%).

#### 1.2 The percentages of Th10 and CD4^+^α-7^+^ T cells are lower and the percentages of Tregs are higher in patients with SCOPD and patients with AECOPD

**Figure 2:**
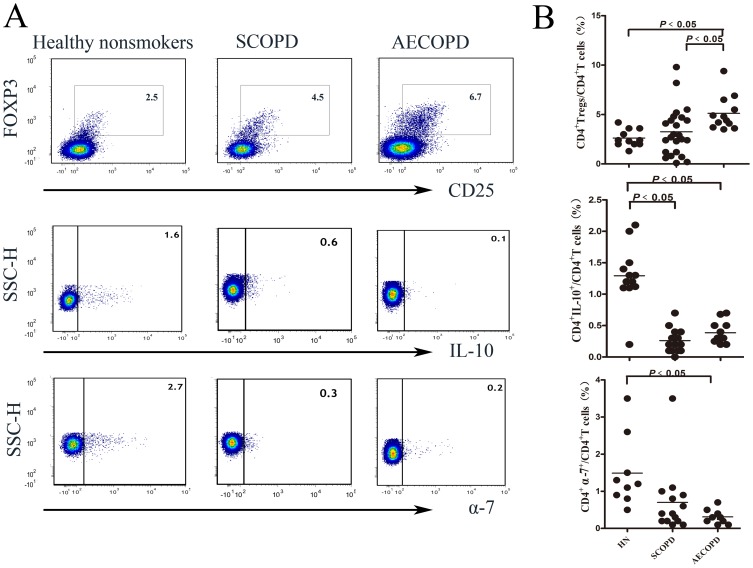
The percentage of Tregs is increased in patients with AECOPD, and the percentages of Th10 and CD4^+^α7^+^ T cells are decreased in both patients with SCOPD and patients with AECOPD. (**A**) Representative flow cytometric dot-plots of Tregs, Th10 and CD4^+^α7^+^ T cells (*Tregs: CD3^+^CD8^−^CD25^+^FOXP3^+^ T cells, Th10: CD3^+^CD8^−^IL-10^+^T cells, and CD4^+^α7^+^ T: CD3^+^CD8^−^α7^+^ T cells*) in healthy nonsmokers, patients with SCOPD and patients with AECOPD are shown. (**B**) Comparisons of the percentages of Th1, Th2 and Th17 cells in healthy nonsmokers, patients with SCOPD and patients with AECOPD *(n = 14, 24, and 14, respectively*) are shown. Horizontal bars indicate the mean value. The comparisons were made using a Kruskal-Wallis one-way ANOVA on ranks.

To investigate the changes in the anti-inflammatory CD4^+^ T cell population in patients with COPD, we detected and compared the percentages of the Tregs (CD3^+^CD8^−^CD25^+^Foxp3^+^), Th10 cells (CD3^+^CD8^−^IL-10^+^), and CD4^+^α-7^+^T cells (CD3^+^ CD8^−^α-7^+^) among the three groups (Figure 2). Due to the limited quantity in some samples, not all CD8^+^ T cell subsets were detected.

Not surprisingly, the percentage of Th10 cells in patients with SCOPD or AECOPD (0.20±0.11%; 0.30±0.10%, respectively) was significantly reduced to approximately one-quarter of that in healthy nonsmokers (1.23±0.54%). Interestingly, the percentage of Tregs was only increased in patients with AECOPD (5.13±1.73%) compared to healthy nonsmokers and patients with SCOPD (2.62±0.86%; 3.08±3.01%, respectively). In contrast, the percentage of CD4^+^α-7^+^ T cells was significantly reduced in patients with AECOPD (0.23±0.11%) compared to healthy nonsmokers (1.48±0.95%). The percentage of CD4^+^α-7^+^ T cells was modestly decreased in patients with SCOPD (0.36±1.16%) compared to healthy nonsmokers, but the difference was not significant.

#### 1.3 Comprehensive analysis of the relative percentages of CD4^+^ T cell subsets

**Figure 3:**
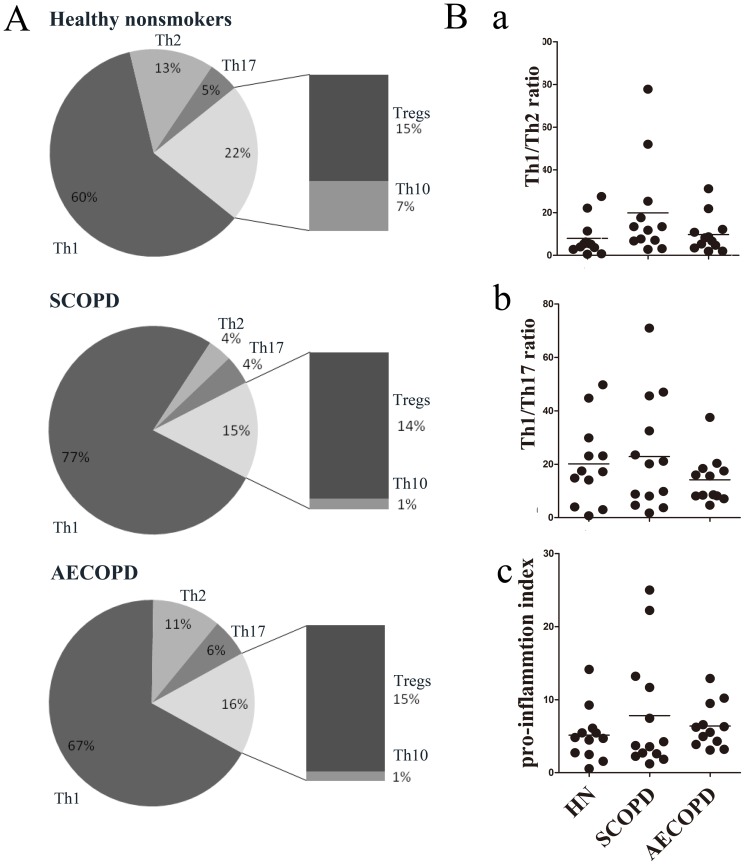
Comprehensive analyses of the relative percentages of CD4^+^ T cell subsets. (**A**) The relative percentages of Th1, Th2, Th17, Treg and Th10 cells in healthy nonsmokers, patients with SCOPD and patients with AECOPD were calculated from their mean values. Anti-inflammatory CD4^+^ T cells consisted of Tregs and Th10 cells. The data are presented in a pie graph. (**B**) The Th1/Th2 ratio (a), Th1/Th17 ratio (b), and pro-inflammatory index (c) in healthy nonsmokers (HN), patients with SCOPD and patients with AECOPD are presented, and the pro-inflammatory index was calculated by dividing the percentage of (Th1+Th2+Th17) by that of (Tregs and Th10).

To clarify the precise changes in CD4^+^ T cell subsets in patients with COPD, we performed a comprehensive analysis of the relative proportion of each subset. As shown in Figure 3, Th1 cells accounted for most of the pro-inflammatory CD4^+^ T cells in both the SCOPD and AECOPD groups (77% and 67%, respectively), and both of these percentages were higher than that observed in healthy nonsmokers (60%). The percentage of Th2 cells was clearly reduced in patients with SCOPD (4%) but were detected at nearly normal levels in patients with AECOPD (11%) compared to healthy nonsmokers (13%). Interestingly, the percentage of Th17 cells was only increased in the AECOPD group (6%) compared to the healthy nonsmoker (5%) and SCOPD (4%) groups. Although the percentage of Tregs was not lower in the SCOPD (14%) and AECOPD (15%) groups compared to healthy nonsmokers (15%), the percentage of Th10 cells was remarkably decreased in both the SCOPD (1%) and AECOPD (1%) groups compared to healthy nonsmokers (7%). As a result, the overall percentage of anti-inflammatory CD4^+^ T cells (Tregs plus Th10 cells) was reduced in patients with AECOPD (16%) and progressively decreased in patients with SCOPD (15%) compared to healthy nonsmokers (22%).

In addition, as indicated in Figure 3B, the Th1/Th2 ratio was notably, although not significantly, elevated in patients with SCOPD (mean value 19.9∶1), but not in patients with AECOPD (mean value 7.9∶1), compared to healthy nonsmokers (mean value 7.8∶1). Interestingly, the Th1/Th17 ratio was modestly increased in patients with SCOPD (mean value 22.9∶1), whereas it was decreased in patients with AECOPD (mean value: 14.2∶1) compared to healthy nonsmokers (mean value: 20.2∶1). In addition, the pro-inflammatory index (<Th1+Th2+Th17> /<Tregs+Th10>) was modestly increased in patients with SCOPD and AECOPD (mean value: 7.8∶1 and 6.3∶1, respectively) compared to healthy nonsmokers (mean value: 5.1∶1).

In total, this comprehensive analysis showed that, compared with healthy nonsmokers, there were relatively more Th1 cells and fewer Th2 and Th10 cells in patients with SCOPD and patients with AECOPD. Additionally, the percentage of Th17 cells was increased in patients with AECOPD, but not in patients with SCOPD. Although the relative percentage of Tregs was normal, the overall levels of anti-inflammatory CD4^+^ T cells were insufficient in these two groups, which lacked Th10 cells. In addition, there was an imbalance in the Th1/Th2 ratio in patients with SCOPD, Th1/Th17 ratio in patients with AECOPD, and the pro/anti-inflammatory ratio in both groups.

### 2 CD4^+^ T cell subsets express different mAChR subtypes

Cigarette smoking is the leading factor contributing to the pathogenesis of COPD. Therefore, we asked whether cigarette smoking caused the imbalance in pro- and anti-inflammatory activity by directly affecting the differentiation of CD4^+^ T cells and Tregs through the up-regulation of the mAchRs. In particular, we examined the mRNA expression of mAchRs and performed differentiation assays with PBT cells obtained from patients with SCOPD and healthy nonsmokers.

#### 2.1 CD4^+^ T cell subsets express different mAChR subtypes

**Figure 4:**
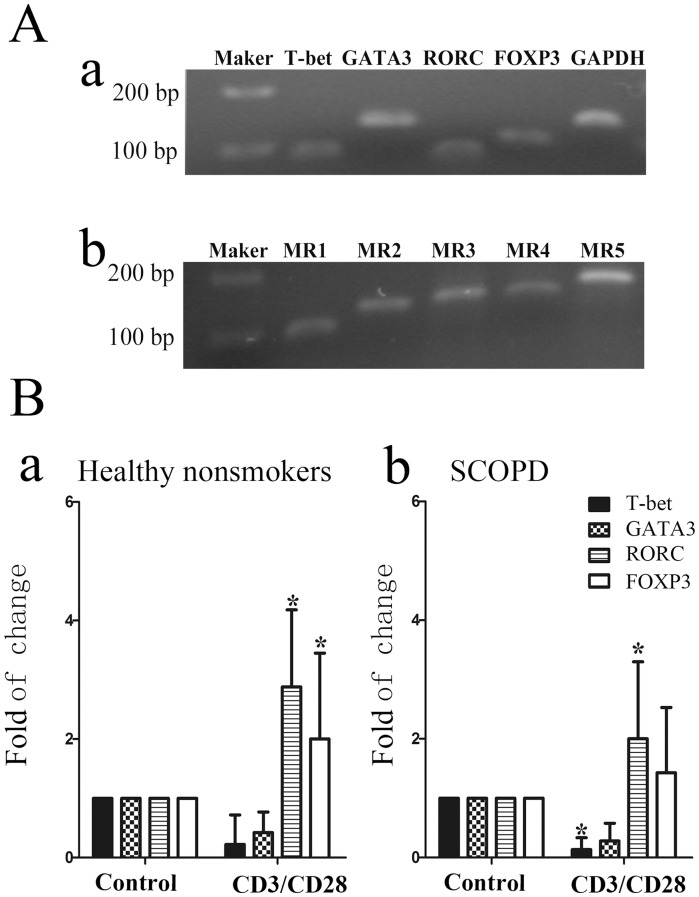
T-bet, GATA3, RORC, FOXP3, and mAChR (MR1–5) mRNA expression in human T cells. (**A**) Nuclear transcription factors (T-bet, GATA3, RORC, and FOXP3) and mAChR (MR1–5) mRNA expression in T cells was confirmed by PCR. (**B**) PBT cells from the healthy nonsmokers (***a***) or patients with SCOPD (***b***) were analyzed either immediately upon isolation or after stimulation with anti-CD3 and anti-CD28 Abs for 5 days, as detailed in the Materials and Methods. The data from 7 independent experiments are expressed as the mean ± SD of the mRNA in question relative to that in unstimulated T cells (control), which is taken as 1. The comparisons were determined by the Kruskal-Wallis one-way analysis of variance on ranks. *P<0.05 compared with the relevant control.

As indicated in Figure 4A, PBT cells expressed MR1-MR5 mRNA, but the RT-PCR results showed that only the expression of MR3, MR4, and MR5 mRNA was relatively stable (data not shown). Compared to unstimulated T cells, the mRNA expression levels of RORC and FOXP3 were significantly increased, whereas those of T-bet and GATA3 were reduced following stimulation with anti-CD3/anti-CD28 Abs, indicating that activation of the TCR facilitates the polarization of naïve T cells to Treg and Th17 cells.

**Figure 5:**
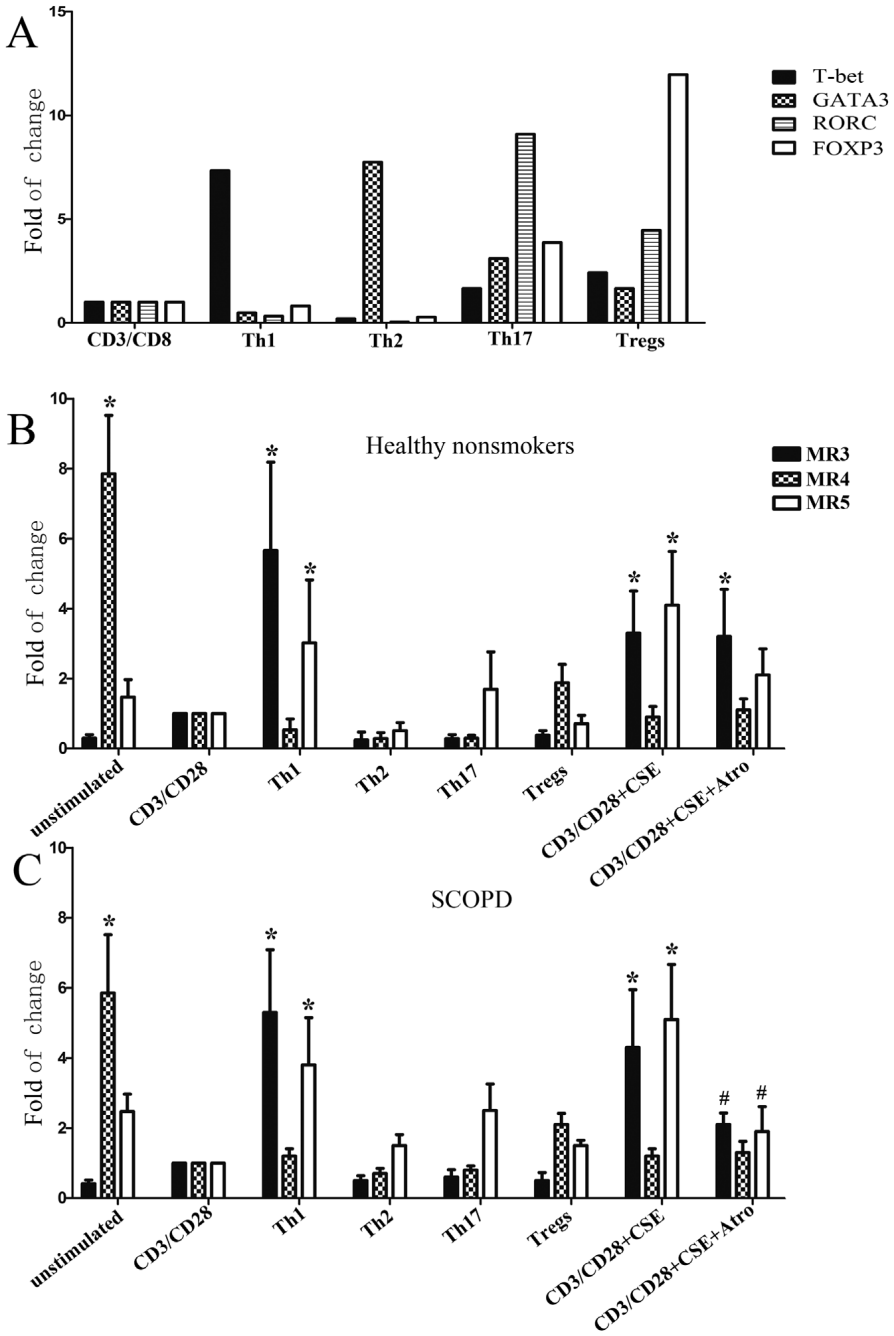
The mAChR subtype changes with polarization into different T cell subsets. PBT cells from healthy nonsmokers (**B**) or patients with SCOPD (**C**) were cultured in conditioned media for polarization towards the Th1, Th2, Th17 and Treg lineages or in the presence of CSE with or without a mAChRs antagonist, and the polarized cells were analyzed by qPCR for the relative levels of mAChR (MR3, MR4, and MR5) mRNA. (**A**) The polarization of T cells was confirmed by the expression of specific nuclear transcription factor mRNA. The data from 7 independent experiments are expressed as the mean ± SD of the mRNA in question relative to that in stimulated T cells (CD3/CD28), which was set as 1. The comparisons were determined by the Kruskal-Wallis one-way analysis of variance on ranks. *P<0.05 compared with the relevant control. ^#^P<0.05 compared with CD3/CD28 plus CSE.

To investigate whether different CD4^+^ T cell subsets express different mAChR subtypes, various exogenous cytokines were used to derive Th1/Th2/Th17/Treg cells. We confirmed the successful polarization of these subsets by their expression of specific nuclear transcription factors, including T-bet, GATA3, RORC, and FOXP3, respectively (Figure 5A). Our data revealed that, in both healthy nonsmokers and patients with SCOPD, the expression of MR3 and MR5 mRNA was significantly up-regulated in Th1 cells (P<0.05) and modestly increased in Th17 cells; MR4 mRNA was modestly increased in Tregs; and there was no significant change in mAchR expression in Th2 cells, although MR5 expression was increased.

#### 2.2 MRs affect the differentiation of CD4^+^T cells

**Figure 6:**
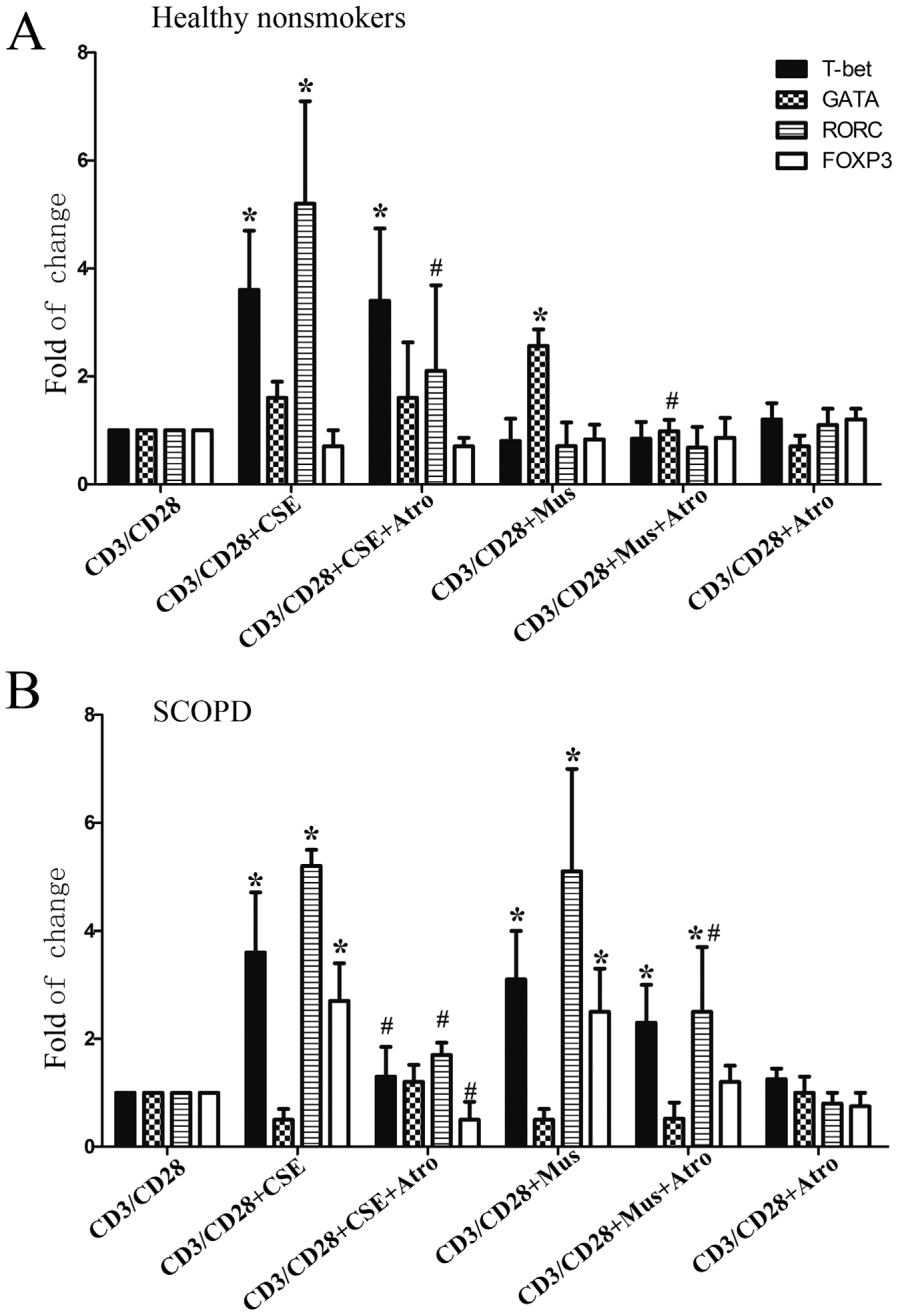
MRs and CSE affect the expression of T cell subset nuclear transcription factors. PBT cells from healthy nonsmokers (**A**) or patients with SCOPD (**B**) were cultured and stimulated with anti-CD3 and anti-CD28 Abs in the presence of CSE, mAChR agonist/antagonist, or combinations of these factors (*as shown in bottom panels*). The agonist and antagonist of mAChRs were muscarine (Mus) and atropine (Atro), respectively. After 5 days in culture, the cells were harvested, and the total RNA was extracted. The expression levels of nuclear transcription factor mRNA, namely that of T-bet, GATA3, RORC, and FOXP3, were detected by qPCR, with GAPDH serving as an internal control. The data from 7 independent experiments are expressed as the mean ± SD of the mRNA in question relative to that in unstimulated T cells (controls), which was set as 1. The comparisons were determined by the Kruskal-Wallis one-way analysis of variance on ranks.*P<0.05 compared with the relevant control. ^#^ P<0.05 compared with CD3/CD28 plus CSE or CD3/CD28 plus Muscarine (Mus).

To assess whether MRs influence the differentiation of CD4^+^ T cells, we performed a differentiation experiment. We found that activation of mAChRs by muscarine significantly enhanced GATA3 mRNA expression (P<0.05) in healthy nonsmokers while up-regulating T-bet, RORC and FOXP3 mRNA expression (P<0.05) in patients with SCOPD, and both effects could be blocked by addition of the mAChR antagonist atropine. Thus, activation of mAChRs promoted the polarization of Th2 cells in healthy nonsmokers and that of Th1, Th17 and Treg cells in patients with SCOPD (Fig. 6A, B).

#### 2.3 CSE affects the mAChR expression and differentiation of CD4^+^ T cells

Our data are the first to show that CSE enhances MR3 and MR5 mRNA expression in T cells in both healthy nonsmokers and patients with SCOPD (P<0.05). Importantly, the increased MR3 and MR5 expression could be blocked by atropine in patients with SCOPD compared to the unchallenged group (Fig. 5B, C). Additionally, CSE significantly increased the T-bet and RORC mRNA expression (P<0.05) in healthy nonsmokers while up-regulating T-bet, RORC and FOXP3 mRNA expression (P<0.05) in patients with SCOPD, and both effects could be blocked by the addition of the mAChR antagonist atropine (Fig. 6A, B). Thus, CSE facilitated the polarization of Th1 and Th17 cells in healthy nonsmokers as well as that of Th1, Th17 and Treg cells in patients with SCOPD via the increased expression of MR3 and MR5 mRNA.

### 3 CSE affects the proliferation and apoptosis of CD4^+^ T cells through MRs

To investigate the effects of cigarette smoking on the proliferation and apoptosis of CD4^+^ T cells and Tregs, we carried out proliferation and apoptosis assays with PBT cells obtained from patients with SCOPD and healthy nonsmokers.

**Figure 7:**
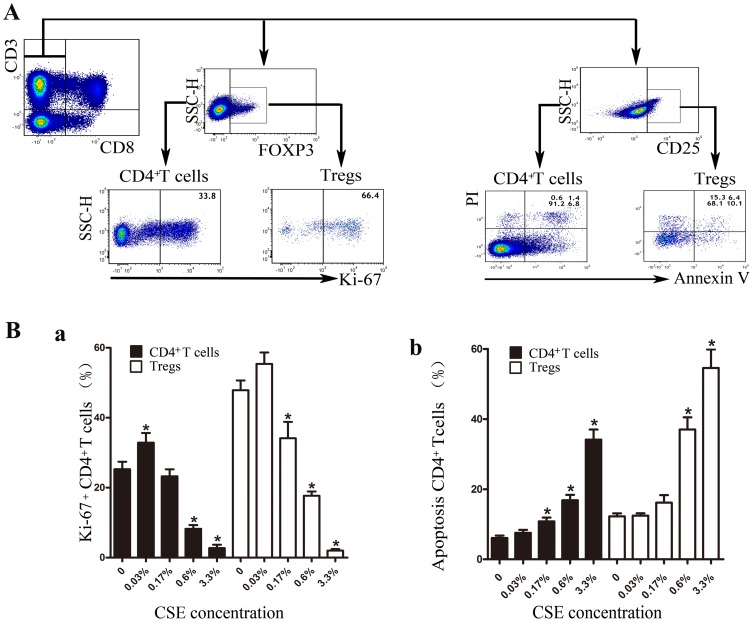
CSE affects the proliferative response and apoptosis of CD4^+^ T cells/Tregs. (**A**) Representative dot-plots showing the analysis strategy. In the proliferation assays, Tregs were gated from CD4^+^T cells (*CD3^+^CD8^−^*) by their positive expression of Foxp3. Then, the expression levels of Ki-67 by CD4^+^T cells and Treg cells were further analyzed. However, in the apoptosis assays, Tregs were gated from CD4^+^T cells (*CD3^+^CD8^−^*) by the high expression of CD25. Then, the apoptosis of CD4^+^T cells and Tregs was further analyzed. (**B**) Various concentrations (0.03%, 0.17%, 0.6% and 3.3%) of CSE were cultured with T cells (PHA and PMA were added in the proliferation assays) for 5 days; then, the expression of Ki-67 was determined by flow cytometry in the proliferation assays (a), while the expression of Annexin V and PI was determined in the apoptosis assays (b). The results are reported as the mean ± SEM from 5 independent experiments. The comparisons were determined by the Kruskal-Wallis one-way analysis of variance on ranks. *P<0.05 compared with control.

First, various concentrations of CSE were added to lymphocyte cultures (Fig. 7). CSE promoted the proliferation of CD4^+^ T cells at a low concentration (0.033% = 0.1 µl of 100% CSE in 300 µl of medium in one well of a 96-well plate), while a high concentration of CSE had the opposite effect (Fig. 7Ba). Therefore, this low concentration of CSE was used in the proliferation assays, and the Ki-67 expression level was used to measure cellular proliferation (Fig. 7A). For the proliferation assays, we also found that various concentrations of CSE affected the apoptosis of CD4^+^ T cells (Fig. 7Bb). CSE facilitated the apoptosis of CD4^+^ T cells even at the concentration of 0.17%, and this pro-apoptotic effect increased at higher concentrations. Therefore, we selected this concentration of CSE to use in the apoptosis assays. PI-negative, Annexin V-positive cells were identified as apoptotic cells (Fig. 7A). In addition, we observed that Treg proliferation and apoptosis were increased compared with those of CD4^+^ T cells. In addition, the effects of CSE on Treg proliferation and apoptosis were similar to those observed in CD4^+^ T cells. However, Tregs appear to be less sensitive to low concentrations of CSE.

#### 3.1 CSE promotes the proliferation of CD4^+^ T cells in healthy nonsmokers and facilitates the apoptosis of CD4^+^ T cells in patients with SCOPD

**Figure 8:**
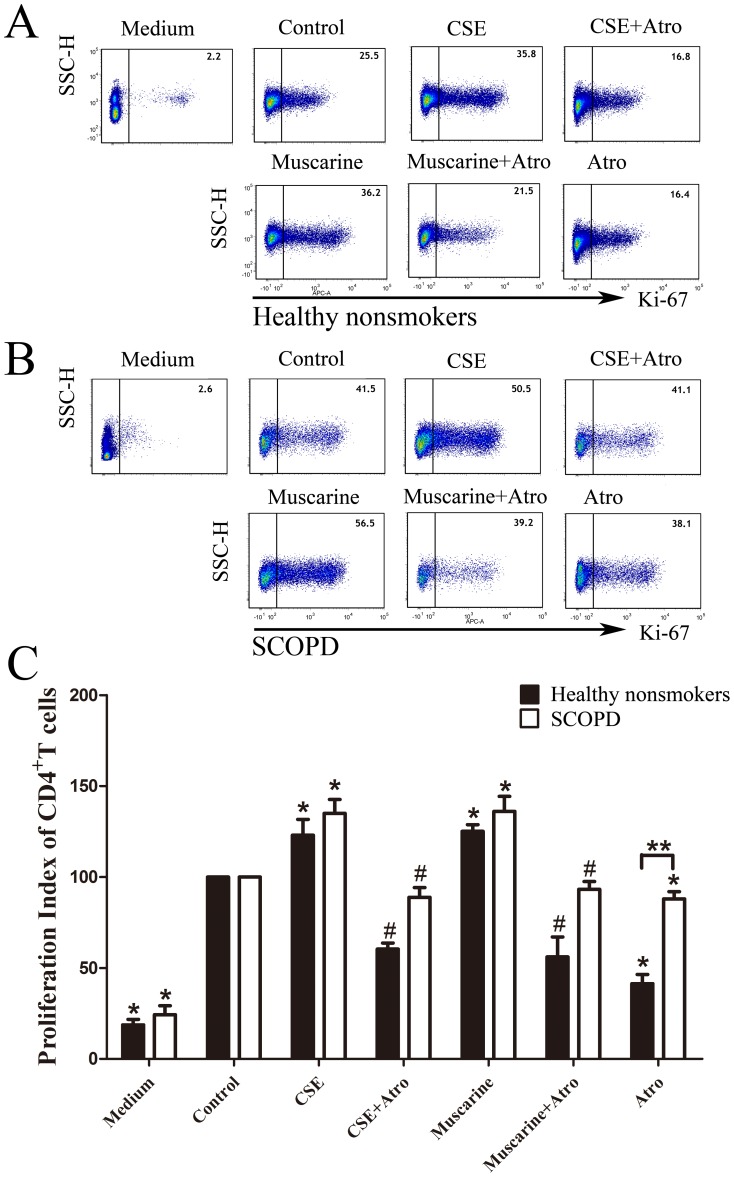
Effects of CSE and MRs on the proliferative response of CD4^+^ T cells. (**A**, **B**) T cells from healthy nonsmokers and patients with SCOPD were cultured in the presence of CSE, mAChR agonist/antagonist, or combinations of these factors (*top panels*) under stimulation with PMA and PHA for 5 days. Ki-67^+^CD4^+^T cells (*excluding Tregs*) were determined by flow cytometry, and the representative flow cytometric dot-plots are shown. (**C**) The proliferation index was calculated by dividing the percentage of Ki-67^+^CD4^+^ T cells of various groups by the percentage of Ki-67^+^CD4^+^T cells stimulated with PMA and PHA alone, and the proliferation index of control was considered to be 100. Comparisons of the proliferation of CD4^+^T cells from the healthy nonsmokers (*solid bars*) or patients with SCOPD (*open bars*) in various trial groups were made; the results are reported as the mean ± SEM from 5 independent experiments. The comparisons were determined by the Kruskal-Wallis one-way analysis of variance on ranks or Mann-Whitney U test. *P<0.05 compared with control, **P<0.05 in the comparison between healthy nonsmokers and patients with SCOPD. # P<0.05 indicates CSE plus Atro compared with CSE alone and Muscarine plus Atro compared with Muscarine alone.

As shown in Figure 8, CSE and mAChR agonists strikingly promoted the proliferation of CD4^+^ T cells (excluding CD3^+^CD8^−^Foxp3^+^ T cells) from healthy nonsmokers and patients with SCOPD. Additionally, this pro-proliferative effect could be neutralized with the mAChR antagonist atropine. We also noted that atropine inhibited the proliferation of CD4^+^ T cells, and this effect was less significant in patients with SCOPD.

**Figure 9:**
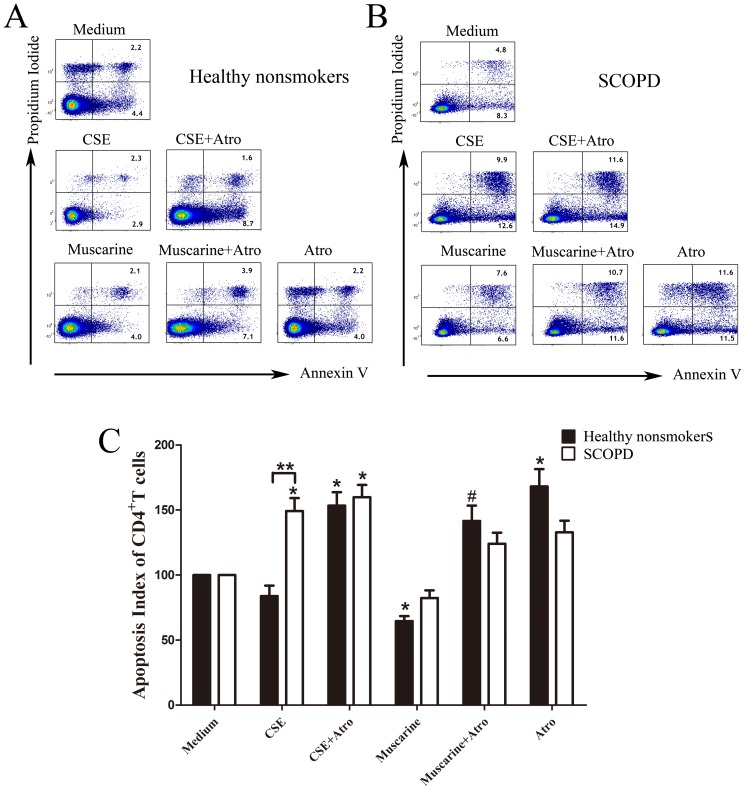
Effects of CSE and MRs on the apoptosis of CD4^+^ T cells. T cells from healthy nonsmokers and patients with SCOPD were cultured in the presence of medium alone, CSE, mAChR agonist/antagonist or combinations of these factors (*top panels*) for 5 days. (**A**, **B**) The representative flow cytometric dot-plots show Annexin V/PI co-staining for the identification of apoptotic CD4^+^ T cells. (**C**) The apoptosis index was calculated by dividing the percentage of apoptotic CD4^+^ T cells in various groups by the percentage of apoptotic CD4^+^ T cells in the medium alone, and the proliferation index of the controls was 100. Comparing the apoptotic CD4^+^T (*excluding Tregs*) cells from healthy nonsmokers (*solid bars*) or patients with SCOPD (*open bars*) in each group, the results were reported as the mean ± SEM from 5 independent experiments. The comparisons were determined by the Kruskal-Wallis one-way analysis of variance on ranks or Mann-Whitney U test. *P<0.05 compared with control, **P<0.05 comparison between healthy nonsmokers and patients with SCOPD. # P<0.05 indicates CSE plus Atro compared with CSE alone and Muscarine plus Atro compared with Muscarine alone.

In regards to apoptosis, CSE facilitated the apoptosis of CD4^+^ T cells (excluding CD3^+^ CD8^−^CD25^++^ T cells) from patients with SCOPD but not healthy nonsmokers. Moreover, the activation of mAChRs by muscarine reduced the apoptosis of CD4^+^ T cells in healthy nonsmokers, and this effect could be completely abolished by treatment with the antagonist atropine (Fig. 9).

In addition, further analysis indicated that CSE promoted the proliferation and inhibited the apoptosis of CD4^+^ T cells in healthy nonsmokers, while modestly facilitating apoptosis in cells from patients with SCOPD (Table 3).

**Table 3:** The net effect of CSE on the survival of CD4^+^ T cells and Tregs.

		Proliferation index	Apoptosis index	Total effect
**Healthy nonsmokers**	**CD4^+^ T cells**	122.9	83.8	Significantly promote proliferation and inhibit apoptosis
	**Tregs**	115.9	90.9	Modestly promote proliferation and inhibit apoptosis
**SCOPD**	**CD4^+^ T cells**	134.9	149.2	Modestly facilitate apoptosis
	**Tregs**	109.3	154.8	Significantly facilitate apoptosis

#### 3.2 CSE significantly promotes the apoptosis of Tregs from patients with SCOPD

**Figure 10:**
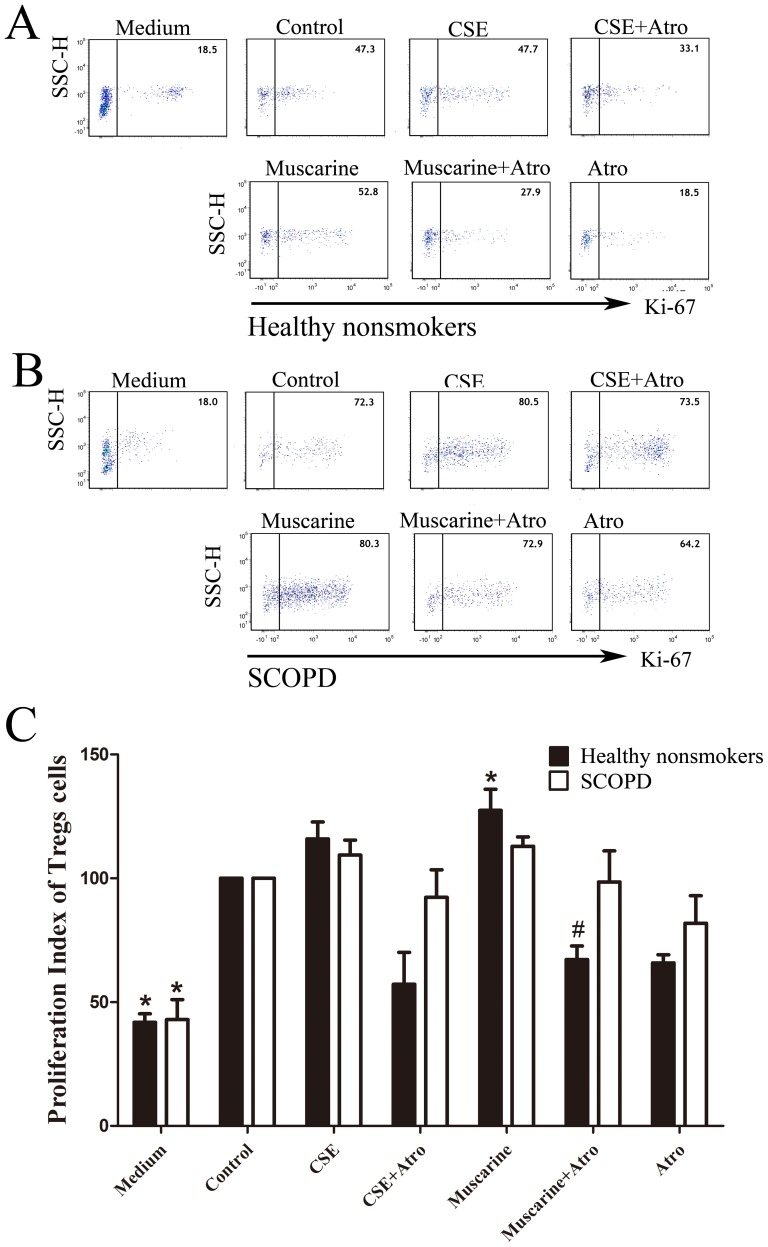
Effects of CSE and MRs on the proliferative response of Tregs. (**A**, **B**) The proliferation of Tregs was further analyzed by flow cytometry, and the representative flow cytometric dot-plots are shown. (**C**) The proliferation index was calculated by dividing the percentage of Ki-67^+^Tregs cells of various groups by the percentage of Ki-67^+^Tregs cells under the stimulation of PMA and PHA alone, and the proliferation index of controls was considered 100. Comparisons of the proliferation of Tregs from the healthy nonsmokers (*solid bars*) or patients with SCOPD (*open bars*) in various trial groups were made; the results were reported as the mean ± SEM from 5 independent experiments. The comparisons were determined by the Kruskal-Wallis one-way analysis of variance on ranks or the Mann-Whitney U test. *P<0.05 compared with control, **P<0.05 for the comparison between healthy nonsmokers and patients with SCOPD. # P<0.05 indicates CSE plus Atro compared with CSE; Muscarine plus Atro compared with Muscarine.

We further analyzed the proliferation of CD3^+^CD8^−^Foxp3^+^ Tregs (Fig. 7A). As indicated in Figure 10, CSE and the mAChR agonist did not significantly affect the proliferation of Tregs in either the healthy nonsmokers or patients with SCOPD. However, stimulation of mAChRs with the agonist muscarine led to the enhanced proliferation of Tregs in healthy nonsmokers but not in patients with SCOPD; furthermore, treatment with the antagonist could counteract this effect.

**Figure 11:**
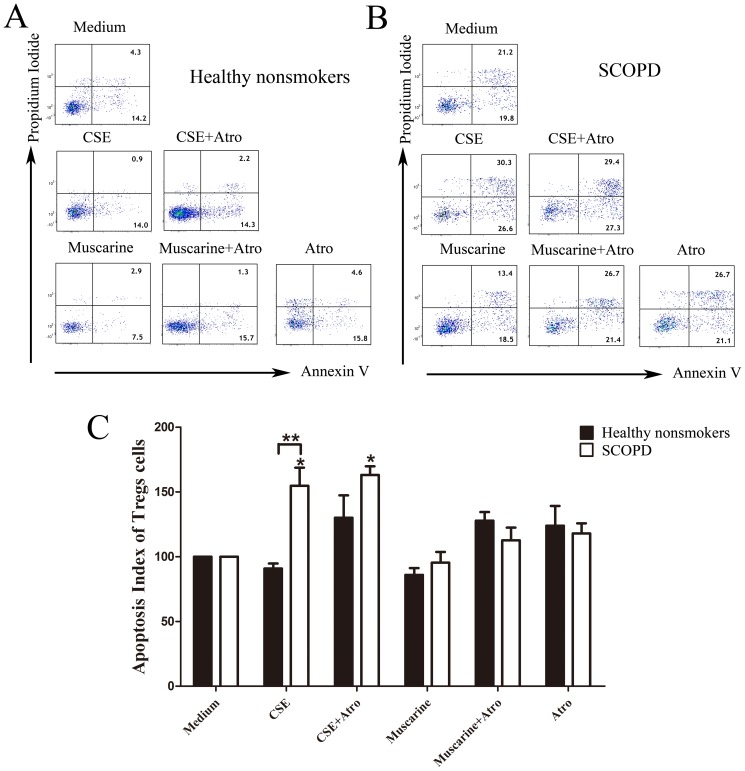
Effects of CSE and MRs on the apoptosis of Tregs. (**A**, **B**) The apoptosis of Tregs was further analyzed by flow cytometry, and the representative flow cytometric dot-plots were shown. (**C**) The apoptosis index was calculated by dividing the percentage of apoptotic Tregs from various groups by the percentage of apoptotic Treg cells in the medium alone, and the apoptosis index of controls was considered to be 100. Comparisons of the apoptosis of Tregs from the healthy nonsmokers (*solid bars*) or patients with SCOPD (*open bars*) in the various trial groups were made. The results are reported as the mean ± SEM from 5 independent experiments. The comparisons were determined by the Kruskal-Wallis one-way analysis of variance on ranks or Mann-Whitney U test. *P<0.05 compared with control, **P<0.05 comparison between healthy nonsmokers and patients with SCOPD. # P<0.05 indicates CSE plus Atro compared with CSE alone and Muscarine plus Atro compared with Muscarine alone.

Considering that the cell membrane must be intact when measuring apoptosis with PI and Annexin V, we used CD3^+^CD8^−^CD25^++^ as an expression signature for Tregs (Fig. 7A). As shown in Figure 11, CSE promoted the apoptosis of Tregs in patients with SCOPD, and the mAChR antagonist could not completely neutralize this effect. Moreover, stimulating mAChRs did not significantly affect the apoptosis of Tregs in these two groups.

Our analysis showed that CSE modestly facilitates the proliferation and inhibits the apoptosis of Treg cells in healthy nonsmokers when used at a low concentration, whereas it significantly promotes apoptosis in cells obtained from patients with SCOPD (Table 3).

## Discussion

### Alterations of T subsets in the three patient groups

Early and recent studies have demonstrated that abnormalities in the immune system play a major role in the development of COPD [18], [19]. In particular, decreased numbers of CD4^+^ T cells and CD8^+^ T cells and a decreased ratio of CD4^+^/CD8^+^ T cells with an abnormal ratio of T helper cells/Tregs and Th17/Tregs have been observed in the peripheral blood in patients with COPD [20]. This reported diversity likely originates from the heterogeneity of peripheral blood T cells in patients with COPD.

Our data indicate that different stages of COPD present disparate alterations in the subsets of T cells and Tregs in the peripheral blood. A remarkable increase in the percentage of Th1 cells with a prominent decrease in the percentage of Th2 cells was observed in patients with SCOPD. In addition, a small increase in the percentage of Th17 cells and an imbalance of Th1/Th2 cells were observed in these patients. Therefore, our data are consistent with previous reports that indicate that COPD is a disease that is predominantly characterized by Th1 responses [21], which is consistent with increased levels of IFN-γ in the airways. Therefore, these features of systemic inflammation in both the local airway and peripheral blood distinguish COPD from asthma, which predominantly involves Th2 responses.

For patients with AECOPD, the inflammatory outburst is mainly due to infection, which leads to a substantial increase in Th1, Th2 and Th17 cells compared to that observed in healthy nonsmokers. Th17 cells play a significant role in pro-inflammatory immune responses against pathogens, and increases in Th1 and Th2 cells facilitate normal immune functions that are similar to those in healthy individuals.

By contrast, we found that the ratio of Th1/Th17 cells was even lower in patients with COPD than healthy controls, which implies that there is an insufficient immune-inflammatory reaction that likely develops into chronic inflammation.

The anti-inflammatory role of the pathogenesis of COPD has attracted increasing attention since the discovery of Tregs. Indeed, different lesion locations in COPD show a variety of changes in the quantity and function of Tregs [22]. Our observations revealed a small increase in the percentage of Tregs, a reduction in the percentage of Th10 cells and a conspicuous decrease in the percentage of Th10 cells in the peripheral blood of patients with SCOPD. In summary, the anti-inflammatory response is decreased in patients with SCOPD compared to healthy nonsmokers. Furthermore, there were more Tregs in patients with AECOPD than in healthy nonsmokers and patients with SCOPD; nevertheless, the percentage of Th10 cells was significantly reduced (accompanied by a decrease in the percentage of α-7+ T cells.) compared to healthy nonsmokers but higher than that in patients with SCOPD.

Our data illustrate the role of Treg cell expansion in AECOPD, which we posit is a compensatory reaction in response to inflammation, as Treg differentiation can be induced by LPS [23] and there is plasticity between Th17 cells and Tregs [24]. Moreover, we demonstrated that the MR4 and MR5 are the major MRs expressed by Tregs and Th17 cells, respectively. This result indicates that ACh could serve as a differentiation regulator in the local inflammatory environment.

Th10 cells can produce IL-10, which decreases the number of Tregs and induces the proliferation of Tr1 cells [25]. Due to technical issues, CD4^+^CD25^+^Foxp3^+^IL-10^+^ T cells could hardly be detected in our experiments; however, the increased percentage of Tregs and the decreased percentage of Th10 cells reveal the possibility of the down-regulation of Treg or Tr1 cell activities in the context of COPD. This result was in accordance with an *in vitro* experiment indicating impaired Treg function in COPD patients [26]. In addition, an imbalance between the anti-inflammatory and pro-inflammatory subsets of Tregs was noted in COPD patients [27].

There are various subtypes of nicotinic acetylcholine receptors expressed on the surface of T cells, and these receptors have a series of complex functions [28]. The high expression of the α-7 subtype can inhibit Th1 and Th17 responses, leading to the induction of Treg in inflammatory bowel disease. In contrast, smoking downregulates the α-7 nicotinic receptor and decreases the levels of IL-10 and Tregs [29], [30]. Both observations show the anti-inflammatory function of the α-7 nicotinic receptor; however, the expression of the α-7 nicotinic receptor in PBT cells of patients with COPD remains unclear. To the best of our knowledge, this is the first report of the expression of the α-7 nicotinic receptor in T cells from patients with COPD. Our results match the features of the T cell subsets of COPD, particularly SCOPD, wherein the Th1 response dominates the microenvironment, which is followed by increasing levels of IFN-γ and IL-12. The subdued Th2 response leads to a reduction in IL-4, which results in lower expression of the α-7 nicotinic receptor. A stronger Th1 response has been correlated with a further reduction in the expression of the α-7 nicotinic receptor, which is more pronounced in patients with AECOPD. In addition, the variation of α-7 T cells is different from that of Tregs and may be related to the diversity in the expression of MRs between Th and Treg cells, although further studies are needed to clarify these findings.

The extent of inflammation depends on the battle between pro-inflammatory and anti-inflammatory forces, and the balance of Th1/Th2, CD4/CD8, and Th17/Treg cells has been proposed as the foundation of the immune response. The emergence of novel technology makes it possible to analyze new subtypes of T cells, such as the nicotinic α-7^+^ T cells. In general, the balance of pro-inflammatory factors and anti-inflammatory factors has been shown to play a major role in inflammation. The balances of the Th1/Th2, CD4/CD8 and Th17/Treg ratios are mainly controlled by Th1, CD8^+^ and Th17 [20], [21], respectively. As shown in Fig. 3, our results suggest that there are minor Th1/Th2 and Th1/Th17 alterations in patients with COPD compared to healthy controls; however, the small number of cases examined may have biased the results.

The pro-inflammatory index is simply defined by the ratio of the percentage of Th1, Th2, and Th17 cells to that of Treg and Th10 cells. We observed an increase in the pro-inflammatory index in patients with AECOPD and in patients with SCOPD, with a higher increase found in patients with SCOPD, which indicates that the characteristics of inflammation in patients with SCOPD are pro-inflammatory and that the Th1 response occupies the dominant role without influencing inflammatory compensation. Nevertheless, an intense Th1 response causes damage to the lung and airway tissue instead of resisting infection. Therefore, inhaled corticosteroids may reduce the local differentiation of CD4^+^ and CD8^+^ T cells and the production of IFN-γ, thereby mitigating local inflammation and suppressing the immune response in patients with SCOPD [5].

### The MR system and the effect of CSE on COPD

Early studies on lymphocytes in the extra neuronal cholinergic system primarily focused on the expression of mAChRs and nAChRs [31], [32], [33]. MR3 has also been reported in patients with COPD [14], [29], but the expression of MRs in subsets of T cells in patients with COPD remains unknown. As shown in Fig. 4, our study is the first to show that MR3 and MR5 are mainly expressed in Th1 and Th17 cells, respectively. In Treg cells, MR4 was dominantly expressed, with a minor expression level of M5. Additionally, we detected the expression of MR5 in Th2 cells. More importantly, CSE not only enhanced the expression of MR3 and MR5 in human lymphocytes but was also inhibited by the M receptor blocker atropine. This blocking effect reached a statistically significant level in patients with SCOPD without exerting an obvious effect on MR4, which differs from murine splenic T cells that mainly expresses MR1 and MR5 [34]. However, MR1, MR3 and MR5 all facilitated cellular proliferation.

In the process T cell subset differentiation, muscarine chiefly induces a Th2 response in healthy individuals that can be blocked by atropine; however, it also induces the proliferation of Th1, Th17 and Treg cells in patients with COPD. Although atropine can restrain this proliferation, an obvious inhibitory effect could only be observed on Th17 cells. This result indicates that the M receptor could also promote the differentiation Th1 and Th17 cells, which forms a positive feedback loop between the M receptor and the induced differentiation of Th cells. Our study is the first to report this finding.

In our study, CSE induced the differentiation and proliferation of Th1, Th17 and Treg cells in both healthy controls and patients with COPD. Furthermore, these processes could be partially inhibited by atropine; the SCOPD group showed a significant inhibition of these processes by atropine. These results verify that the expression of MR1 and MR5 can be induced by CSE, thus promoting the differentiation of Th1 and Th17 cells. Hence, for the first time, we report positive feedback between Th1/Th17 cells and the expression of MR1/MR5 in patients with COPD. In both trials, there were no significant alterations in Th2 cells or MR4 expression, which is consistent with our previous report of the use of an MR3 receptor blocker, tiotropium bromide, to treat patients with COPD [35]. The proportion of CD4^+^ and CD25^+^ decreases remarkably after the blockage of MR3, i.e., the differentiation of Th1 and Th17 was inhibited, which is in line with recent reports [36].

Our results disagree with those in murine splenic T cells [34], which is mainly due to differences between species. Murine spleens mostly express MR1 and MR5 with scarce expression of MR3. Our data verify that Th1 cells in humans express MR3 and MR5, whereas the expression of MR3, MR4 and MR5 was rare in Th2 cells. Following stimulation by muscarine, the Th2 response becomes dominant, although this effect could be inhibited by atropine. These observations are in agreement with earlier reports showing dominant expression of Th2 cells in the fetus of women who smoked during pregnancy [37]. However, the increases in the proportion of Th1, Th17 and Treg cells caused by stimulation with muscarine seemed to be more prominent in patients with COPD, which was different from that observed in healthy groups. Based on our results, this difference may be due to lack of the α7 nicotinic receptor in patients with COPD.

### Influence of CSE on the proliferation and apoptosis of CD4^+^ Th/Tregs

The data presented in Fig. 8 show that muscarine significantly promoted the proliferation of CD4^+^T cells in both healthy nonsmokers and patients with SCOPD by activating the mAChRs, and the pro-proliferation effect could be neutralized by atropine. Thus, activation or hyperfunction of mAChRs leads to the enhanced proliferation of CD4^+^ T cells. CSE could similarly facilitate the proliferation of CD4^+^T cells in both groups, and the pro-proliferation effect could also be counteracted by atropine, indicating that CSE may affect the proliferation of CD4^+^T cells through the muscarine system. Previous research demonstrated a cholinergic system in lymphocytes, and muscarine was shown to promote the proliferation of the CD4^+^ T cells by enhancing the production of IL-2 [38]. Subsequently, a study reported that both muscarinic and nicotinic receptors regulate the proliferation and apoptosis of CD4^+^ T cells in animals and humans [39], [40]. With respect to the current knowledge, our study is the first to show that CSE could facilitate the proliferation of CD4^+^ T cells through up-regulating the muscarine system, thereby aggravating the inflammation present in the airways of patients with COPD.

Our data also show that muscarine could inhibit the apoptosis of CD4^+^T cells in healthy nonsmokers (Fig. 9), while atropine had an opposing pro-apoptotic effect, demonstrating that activation of mAChRs could reduce the apoptosis of CD4+ T cells. Interestingly, CSE promoted the apoptosis of CD4+T cells from patients with SCOPD but not in healthy nonsmokers, and this effect could not be neutralized by atropine. CSE-induced apoptosis is potentially mediated by a variety of mechanisms, including increased oxidative stress, Bax protein accumulation, mitochondrial dysfunction, mitochondrial cytochrome c release and NF-κB inhibition [41], [42], [43], [44], [45], [46]. It is unknown why CD4^+^ T cells from healthy nonsmokers and patients with SCOPD react differently to CSE with respect to apoptosis. However, we speculate that CD4^+^ T cells may present alterations during the development of the disease, such as enhanced reactivity to oxidative stress, up-regulation of receptors that are sensitive to apoptosis or down-regulation of receptors with inhibitory functions, resulting in a distinct apoptotic response.

We found that the MR system had a pro-proliferative effect on Tregs in healthy nonsmokers, which could be blocked by atropine (Fig. 10), suggesting a reliance of Tregs on mAChRs. However, this phenomenon was not obvious in patients with COPD, who showed no responses to muscarine and atropine; the reason for these differences is not yet known but is potentially related to the reduction of nicotinic receptor α-7 expression. Additionally, CSE only modestly, but not significantly, enhanced the proliferation of Tregs in healthy nonsmokers and patients with SCOPD, and this effect could not be offset by atropine. As a result, the MR system may not have pro-proliferative effects on Tregs in patients with SCOPD.

Our results imply that neither muscarine nor atropine significantly affect the apoptosis of Tregs (Fig. 11). Therefore, the muscarine system may not be involved in the apoptosis of Tregs. However, CSE robustly promoted the apoptosis of Tregs in patients with SCOPD, and atropine could not completely reverse this effect, indicating that CSE induces the apoptosis of these cells without affecting the muscarine system. The following potential mechanisms may explain these findings: 1) oxidative stress, 2) poisonous substances contained in CSE, and 3) abnormal alterations in the Tregs of patients with SCOPD. Considering that apoptosis of Tregs from healthy nonsmokers was not affected by CSE, there may be substantial changes in these cells during the development of COPD. Taken together, our data indicate that CSE modestly facilitates the proliferation and inhibits the apoptosis of Tregs in healthy nonsmokers, but it also significantly promotes apoptosis in cells from patients with SCOPD. Further studies are needed to clarify the mechanisms underlying the different reactions to CSE in different populations.

In summary, our current research identifies an imbalance of pro/anti-inflammatory CD4^+^ T subsets in patients with COPD, with increased percentages of Th17 cells (only in AECOPD) and Th1 cells and reduced percentages of Th2 cells (only in SCOPD) and Th10 cells, as well as a reduced quantity and impaired capacity of Tregs. Our study is the first to report the expression of MRs in T cell subsets from the peripheral blood in these three patient groups, and our results reveal a positive feedback loop between the MR and the induced differentiation of Th cells. We also speculate there is a lack of α-7 nicotinic receptor expression in patients with COPD. The different reactions to smoke observed in our study indicate that there are differences in the genetic sensitivity between healthy individuals and patients with COPD, although further studies need to be conducted to acquire a full explanation of these interesting phenomena.
